# Nuclear glycogenolysis modulates histone acetylation: a novel mechanism of epigenetic regulation in cancer

**DOI:** 10.1186/s40880-019-0420-6

**Published:** 2019-11-09

**Authors:** Xifeng Dong

**Affiliations:** Department of Hematology, General Hospital, Tianjin Medical University, Tianjin, 300052 P. R. China

Cancer cells, as compared to normal cells, have their own metabolic alterations [1] which are essential for their survival and proliferation as they have higher energy production [2], maintenance for redox potential [3], and anabolic pathway induction [4]. Thus, metabolic reprogramming has been considered as a potential therapeutic target of cancer. In recent years, several studies have shown evidences that metabolic reprogramming is regulated by the genetic events of cancer cells. However, as the metabolites of the cancer cells also simultaneously affect epigenetic events, these make cancer cells dependent on certain metabolic-related substances, such as lipids and glutamine [5].

Glycogen, a multibranched polysaccharide of glucose, is the main storage form of glucose that can be broken down to yield glucose molecules when cells need energy. It is found in most normal tissues and accumulated in specific subcellular organelles. Several studies have reported that glycogen was elevated in multiple cancer cell lines, including lung [6], breast [7], and colorectal cancer [8]. Additionally, hypoxia, a key characteristic of tumor mass, can enhance carcinogenesis, suppress reactive oxygen species levels and inhibit senescence via inducing glycogenolysis in breast [7] and colorectal cancer [8]. These studies suggested that glycogen can serve as an important energy source in cancer cells to enable survival and proliferation under hypoxia. However, aside from being an energy source, the potential roles of glycogen in cancer are still unclear.

In a recent study published in *Cell Metabolism*, entitled “Nuclear Glycogenolysis Modulates Histone Acetylation in Human Non-Small Cell Lung Cancers”, Sun et al. [9] demonstrated that nuclear glycogen metabolism plays a critical role in providing substrates for histone acetylation in non-small cell lung cancer (NSCLC) and further explored how NSCLC cells can perturb this pathway to support proliferation under hypoxia. By using nuclei preparations and isotope tracers, the authors observed that nuclear glycogen was de novo synthesized in NSCLC cell lines to promote pyruvate pool formation for histone acetylation; thereby promoting the development and progression of NSCLC. Mechanism studies have shown that NSCLC cell lines can accumulate nuclear glycogen by down-regulating marlin, an E3 ubiquitin ligase that could enhance glycogenolysis, which were validated in animal xenograft models and 50 paired human NSCLC tissues and normal lung tissues. These data showed that nuclear glycogen is not only a polymer compound that serves as a form of energy storage but also a signaling molecule in NSCLC.

One of the innovations of this study is that the authors used a novel combination method, combine isolation methods with highly sensitive tracking technologies, to detect the metabolism of specific organelles. In addition, this study demonstrates that nuclear glycogen of NSCLC cells provides a carbon source for histone acetylation, and E3 ubiquitin ligase and glycogen phosphorylase ubiquitination is involved in this metabolic process. In other words, the downregulation of glycogenolysis due to decreased malin and nuclear glycogen phosphorylase expression further leads to a lack of substrate for histone acetylation and ultimately results in an alteration of the epigenetic landscape seen in NSCLC. Based on these findings, the authors proposed a new concept that E3 ubiquitin ligase can serve as a signaling molecule by regulating glycogen metabolism in the nucleus of NSCLC cells.

Mutations in metabolic enzymes result in the upregulation of oncometabolites, for instance, mutations in isocitrate dehydrogenase result in excess production of 2-hydroxyglutarate [10]. Nearly all of these oncometabolites have been reported to cause cancer malignancy by inhibiting DNA and/or histone demethylases [5]. Aberrant histone acetylation is a hallmark in multiple cancers, especially NSCLC. Vorinostat, a histone deacetylase inhibitor that can increase histone acetylation, has been demonstrated to have anti-cancer activity in NSCLC [11]. However, the mechanisms by which oncometabolites affect histone acetylation remains unclear. In normal cells, nuclear localization of some metabolic enzymes, such as adenosine triphosphate citrate lyase, pyruvate dehydrogenase, and acetyl-coenzyme A synthase, can supply histone acetylation [12–14]. Although these metabolic enzymes have been shown to promote histone acetylation by using pyruvate and acetate, the origin of their compartmentalized substrates still remains unknown. The authors have demonstrated that glycogen contributes to the formation of pyruvate and acetate pool in the nucleus. This study reveals an underlying molecular mechanism of an observation that has been in existence for more than 70 years; thereby redefining the role of nuclear glycogen in cancer biology and provided a new therapeutic target for NSCLC.

## Data Availability

Not applicable.

## Abbreviation

NSCLC: non-small cell lung cancer
